# The physical chemistry of high-sensitivity differential scanning calorimetry of biopolymers

**DOI:** 10.1007/s40828-016-0038-0

**Published:** 2016-12-27

**Authors:** Stephen Leharne

**Affiliations:** Faculty of Engineering and Science, University of Greenwich, Pembroke, Chatham Maritime, Kent, ME4 4TB UK

**Keywords:** Calorimetry, Thermodynamics, Biomolecules

## Abstract

High-sensitivity differential scanning calorimetry (HSDSC) is widely used to examine the thermal behaviour of biomolecules and water-soluble polymers in aqueous solution. The principal purpose of this manuscript is to examine the thermodynamic basis for the signals obtained using HSDSC. It is shown that a combination of the van’t Hoff isochore and Kirchhoff’s equation are all that is necessary to simulate and curve fit the HSDSC output obtained for the thermally induced unfolding of the protein ubiquitin. The treatment is further developed to show how the temperature dependence of the heat capacity change of unfolding, multiple sequential transitions, and protein dissociation can be incorporated into the thermodynamic description of protein unfolding and how these factors in turn affect the HSDSC signal.

## Introduction

High-sensitivity differential scanning calorimetry (HSDSC) is widely employed for the study—in aqueous solution—of the thermodynamic parameters associated with processes initiated either by an increase in temperature (up-scan) or by a decrease in temperature (down-scan). Small molecular mass molecules cannot be examined by HSDSC unless they form aggregate structures showing intermolecular co-operation. On the other hand, biopolymers in aqueous solution, such as proteins, which are cooperatively stabilised by numerous weak forces, can be examined by HSDSC.

Typically HSDSC can be used to examine:

1. Transitions from the physiologically active native form of a protein through intermediate partially unfolded states to the final denatured form of the protein. Very often, such a process is characterised by minimally populated intermediate states and thus approximates to a two-state transition between the initial native form and the final denatured form of the protein [1].
2. Thermally induced co-operative transitions in molecular assemblies of phospholipids, such as multi-lamellar liposomes [2].
3. Melting transitions in DNA and oligonucleotides [3].

In HSDSC, the specific heat of an aqueous system is measured as a function of temperature. For an aqueous solution of a bio-polymer, the apparent specific heat of the solute (*S*_2_) is given by the following expression [1]:

1 $$S_{2} = S_{1} + \frac{1}{{w_{2} }}(S - S_{1} )$$

where *S* is the specific heat of the solution, *S*_1_ is the specific heat of the solvent, and w_2_ is the weight fraction of the solute. Because the quantity (*S* − *S*_1_) is usually very small, a differential mode of measurement [solvent (reference cell) versus solvent plus solute (sample cell)] has to be used. Indeed, given that a major portion of the specific heat change is due to the heating and cooling of the solvent (usually water which has a large heat capacity), it is essential to have a differential arrangement, so that phase transitions in the solute can be observed.

## HSDSC signals and their interpretation for protein unfolding

DSC instruments measure the power required to maintain the temperature of a sample placed in a designated sample cell at (or close to) the same value as that of a reference cell containing the identical aqueous solvent, but no sample molecule, as the overall temperature of the system, is altered. The cells are located within an adiabatic vacuum chamber. The raw instrumental output conventionally shows power as a function of temperature. To extract data that have more thermodynamic significance, the axes of the trace output are transformed. Power is converted to a molar excess heat capacity using the formula:

2 $$\frac{{{\text{d}}q_{p} }}{{{\text{d}}t}}.\frac{1}{\sigma M} = C_{p,xs}$$

where *q*_*p*_ is the heat absorbed at constant pressure; *t* is time, the derivative d*q*_*p*_/d*t* represents power; *σ* is the scan rate (d*T*/d*t,* where *T* is temperature); and *M* is the number of moles of sample in the sample cell.

A typical DSC experiment normally involves at least two scanning runs. One scan consists of a baseline scan, wherein the sample cell and reference cell both contain the blank aqueous solvent. The second scan is a scan of the solvent (reference cell) against the solvent plus solute (sample cell). The baseline scan is then subtracted from the sample scan.

**Fig. 1:**
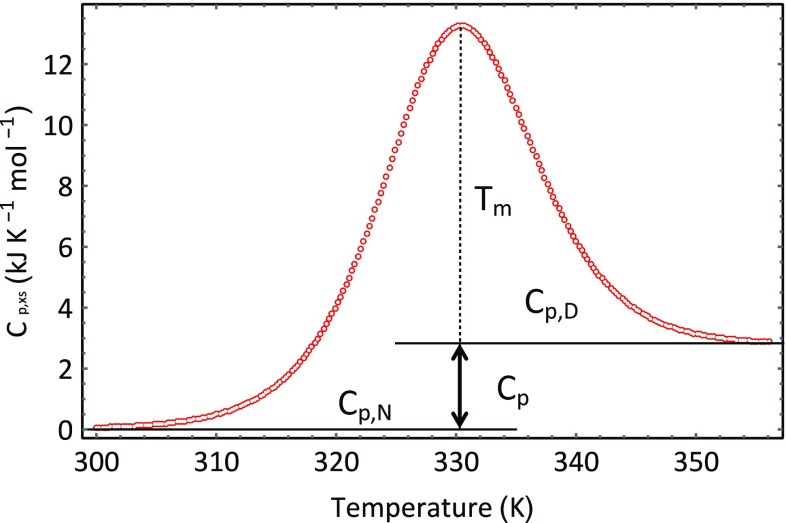
Data obtained for a 5 mg cm^−3^ solution of ubiquitin at a pH of 2

Figure 1 provides a typical example of an HSDSC trace of the excess heat capacity (the heat capacity difference between the sample and reference cells) as a function of temperature. The signal shown in Fig. 1 was obtained for the protein ubiquitin in buffer solution, at a pH of 2.

Proteins undergo denaturation on heating. The process involves a transition from the physiologically active compact folded form to the normally physiologically inactive unfolded form. Native protein structures in aqueous solution are cooperatively stabilised by numerous intramolecular forces. Disruption of these forces requires an endothermic enthalpy change. The favourable free energy contribution to denaturation is provided by the entropy change that arises from the increased conformational freedom available to the unfolded protein and the increased number of ways of partitioning the increased thermal energy.

**Fig. 2:**
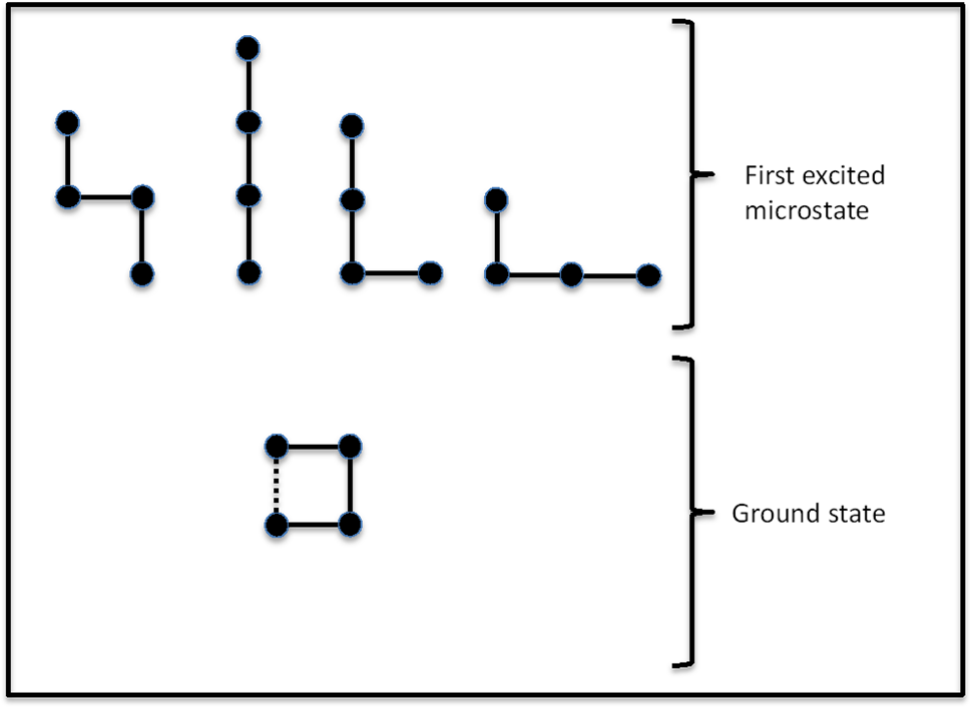
Four-bead model for molecular unfolding. (Redrawn from Dill and Bromberg [4])

A simple pedagogic model of thermally induced unfolding has been described by Dill and Bromberg [4]. Consider a four-bead molecular chain, as shown in Fig. 2. In this model, the ground state is characterised by a compact molecular structure that is held together by an intramolecular bond (the dashed line) between the chain-ends. The first excited microstate is fourfold degenerate—i.e., there are four different unfolded molecular conformational structures, of equal energy that the molecule can adopt. The fractional occupancy of the two different energy states and its functional relationship with temperature can be calculated using the Boltzmann distribution equation:

3 $$\frac{{n_{1} }}{{n_{0} }} = \frac{{g_{1} e^{{ - \frac{{\varepsilon_{1} }}{kT}}} }}{{g_{0} e^{{ - \frac{{\varepsilon_{0} }}{kT}}} }}.$$

The subscripts 0 and 1 denote the ground state and first excited microstate, respectively; *n* is the number of molecules in a particular state; *g* is the degeneracy of that state with *g*_*o*_ = 1 and *g*_1_ = 4;  *ε* is the energy of the state; *k* is the Boltzmann constant; and *T* is the absolute temperature.

Using the mass balance expression *n* = *n*_0_ + *n*_1_ and Δ*ɛ* = *ɛ*_1_ − *ɛ*_0,_ we can rewrite Eq. 3 as

4 $$\frac{{n - n_{0} }}{{n_{0} }} = 4e^{{ - \frac{\Delta \varepsilon }{kT}}}$$

which gives the following expressions for the fraction of molecules in the ground state:

5 $$\frac{{n_{0} }}{n} = \frac{1}{{1 + 4e^{{ - \frac{\Delta \varepsilon }{kT}}} }}$$

and fraction of molecules in the excited state:

6 $$\frac{{n_{1} }}{n} = \frac{{4e^{{ - \frac{\Delta \varepsilon }{kT}}} }}{{1 + 4e^{{^{{ - \frac{\Delta \varepsilon }{kT}}} }} }}.$$

**Fig. 3:**
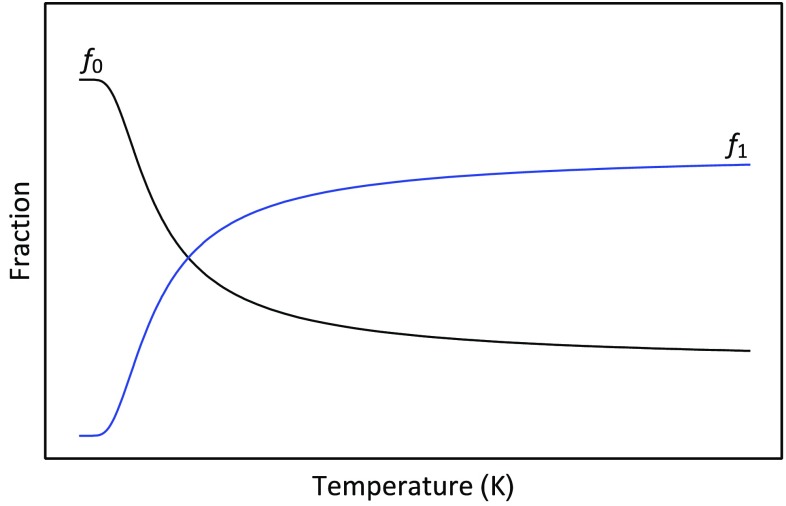
Changing composition of the four-bead molecular system as a function of temperature. The fraction of molecules in the ground state is designated *f*_0,_ and the fraction of molecules in the first excited state is designated *f*_1_

The temperature dependence of the composition of the system is shown in Fig. 3. This system is an example of a two-state system, i.e., a system within which only two states are significantly populated. At low temperatures, the ground state form predominates. The enthalpy of intramolecular binding is key to this predomination. However, as the temperature rises, the excited state becomes increasingly populated, thereby demonstrating the increasingly important entropic contribution of conformational variety to the system. The statistical thermodynamic description of protein unfolding is far more complex than the four-bead molecular model, but the model does encapsulate one of the reasons as to why proteins unfold upon heating to moderately high temperatures—the large number of excited state conformers. Moreover, just like the model, protein unfolding is, very often, a two-state process. As a consequence, the signal shown in Fig. 1 can be interpreted as showing how the thermal history of the system reflects the changing composition of the aqueous protein system as temperature increases. At low temperatures, the compact native form predominates as the temperature is increased some molecules begin to unfold. The fraction of molecules that have unfolded multiplied by the enthalpy of the unfolding transition provides the basis of the heat signal. Since the enthalpy change is endothermic, the temperature of the sample cell becomes lower than that of the reference cell; and thus, the instrument measures the power needed to raise the temperature to compensate for the temperature difference. This, as we have shown, is easily converted into a molar excess heat capacity.

## The DSC signal and initial data analysis

There are several features of the DSC signal, as shown in Fig. 1, which require comment. The transition from the compact physiologically active form of the protein to the more open unfolded physiologically inactive molecular form is shown as an increase in heat capacity of the system, going through a maximum at a temperature designated as *T*_m_ and then decreasing to a final higher final heat capacity value. The initial low temperature portion of the scan represents the heat capacity of the native form of the protein in aqueous solution (denoted as *C*_*P,N*_). The high-temperature portion of the scan shows the heat capacity in aqueous solution of the unfolded form of the protein (denoted as *C*_*P,D*_). In this scan, both heat capacities are assumed to be invariant with the temperature over the temperature range of the experimental run, so the heat capacity change on unfolding given by the expression Δ*C*_*p*_ = *C*_*p*,*D*_ − *C*_*p*,*N*_ is a constant. Formally, the molar heat capacity is the amount of heat energy required to the raise the temperature of 1 mol of substance through 1 K. At the molecular level, the additional heat energy is distributed among the various degrees of freedom and partitioned variously between kinetic energies—including vibrational, rotational, and translational transitions and potential and potential energies—including stretching and bending of molecular bonds [5].

The existence of the heat capacity change indicates that both enthalpy and entropy are functionally dependent upon temperature. If Δ*C*_*p*_ is constant, then we can write:

7 $$\left( {\frac{\partial \Delta H}{\partial T}} \right)_{p} = \Delta C_{p} \underrightarrow {\text{Integration}}\,\,\Delta H(T) = \Delta H(T_{\text{ref}} ) + \Delta C_{p} (T - T_{\text{ref}} ).$$

Using the second law of thermodynamics, we get a similar expression for the entropy change:

8 $${\text{d}}\Delta S = \frac{{{\text{d}}q_{\text{rev}} }}{T} = \frac{{{\text{d}}\Delta H}}{T} = \frac{{\Delta C_{p} }}{T}{\text{d}}T\,\,\underrightarrow {\text{Integration}}\,\,\Delta S(T) = \Delta S(T_{\text{ref}} ) + \Delta C_{p} \ln \left( {\frac{T}{{T_{\text{ref}} }}} \right).$$

There are several reasons why the overall heat capacity change for protein unfolding increases. These include the exposure of hydrophobic amino acid side chains buried in the core of the native form of the protein to water when the protein molecule unfolds. For example, Connelly and Thomson [6] noted that the dissolution of aliphatic and aromatic hydrocarbons in water invariably leads to an increase in heat capacity. However, there are likely to be other contributions, such as an increase in easily excitable vibrational modes upon unfolding.

**Fig. 4:**
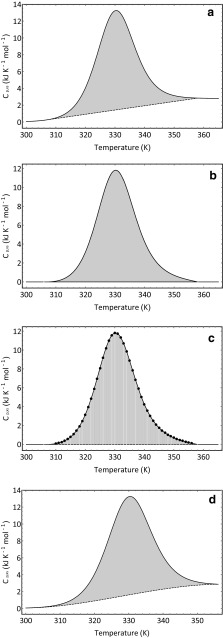
Integration of the peak area. **a** Baseline is constructed so as to connect the start of the transition and the end of the transition. **b** Baseline is subtracted from the data. **c** Area under the peak is divided up into evenly spaces segments that are then use to calculate the area either using the trapezoidal rule or the Simpson’s rule. **d** Baseline fitted to the pre- and post-transitional portions of the signal using a cubic polynomial (see text for details)

The enthalpy of denaturation can be obtained from the experimental data by integration of the peak area. This value is referred to as the calorimetric enthalpy (Δ*H*_cal_). To obtain the peak area, we must first draw a baseline to the data. In the example shown in Fig. 4a, a straight line is drawn from what is judged to be the start of the transition to the adjudged end of the transition. Once satisfied that the baseline satisfactorily joins the onset and termination of the thermal transition, it is then subtracted from the HSDSC data (Fig. 4b) to leave a transitional profile with a flat baseline. The resultant signal can then be divided up into evenly spaced segments. The area of the individual segments can then be computed either by the trapezoidal rule or by the Simpson’s rule and then summed to give the integrated peak area (Fig. 4c).

The trapezoidal rule equation is

$${\text{Area}} = [f(x_{0} ) + 2f(x_{1} ) + 2f(x_{2} ) \rightleftharpoons 2f(x_{n - 1} ) + \, f(x_{n} )]\frac{\text{Interval}}{2}$$

whilst the Simpson’s rule equation is

$${\text{Area}} = [f(x_{0} ) + 4f(x_{1} ) + 2f(x_{2} ) + 4f(x_{3} ) \rightleftharpoons 2f(x_{n - 1} ) + \, f(x_{n} )]\frac{\text{Interval}}{3}.$$

Using the trapezoidal rule, Δ*H*_cal_ was found to be 198 kJ mol^−1^.

Straight-line baselines are convenient and easily drawn but do not necessarily reflect the true geometry of the underlying baseline. Other baselines can be fitted. In Fig. 4d, the pre- and post-transitional portions of the signal are fitted to a cubic polynomial of the form: $f(x) = ax^{3} + bx^{2} + cx + d.$ Other functions can be used—for example, quartic order polynomials or cubic splines—which may represent the underlying baseline better. Yet, normally, area integration using a straight-line baseline provides values not too dissimilar to values obtained using other baseline functions.

## Can we use thermodynamics to examine the HSDSC signal?

Thermodynamic information may be obtained from the signal if it can be established that the signal was obtained under conditions of thermodynamic equilibrium. Thermodynamic control of the thermal processes observed in the calorimeter may be established by investigating the reversibility of the system. If the system either reproduces the same trace on rescanning or produces an identical trace on cooling the application of thermodynamic relationships to aid our understanding of the HSDSC trace is justified. A further test of the applicability of thermodynamics is to examine the HSDSC signals for scan rate dependence. Parameters measured for processes under thermodynamic control show no scan rate dependence [7].

For proteins where unfolding is a two-state process the fraction of protein in the native state is given by the following expression that is analogous to the expression for the ground state in the four-bead model:

9 $$f_{N} = \frac{1}{{1 + e^{{ - \frac{\Delta G}{RT}}} }}$$

and similarly for the denatured state

10 $$f_{D} = \frac{{e^{{ - \frac{\Delta G}{RT}}} }}{{1 + e^{{ - \frac{\Delta G}{RT}}} }}.$$

The energy difference between the two states is the free energy of denaturation (Δ*G*). The ratio of the fraction of the denatured protein to the fraction of native protein is equal to the ratio of the concentrations of the denatured ([*D*]) and native protein ([*N*]):

11 $$\frac{{f_{D} }}{{f_{N} }} = \frac{{\frac{[D]}{{P_{t} }}}}{{\frac{[N]}{{P_{t} }}}} = \frac{[D]}{[N]}$$

where *P*_*t*_ is the total concentration of protein.

Using Eqs. 7 and 8, the ratio is also equal to the following expression:

12 $$\frac{{f_{D} }}{{f_{N} }} = \frac{{\frac{{e^{{ - \frac{\Delta G}{RT}}} }}{{1 + e^{{^{{ - \frac{\Delta G}{RT}}} }} }}}}{{\frac{1}{{1 + e^{{^{{ - \frac{\Delta G}{RT}}} }} }}}} = e^{{ - \frac{\Delta G}{RT}}} .$$

However, from fundamental thermodynamics, we know that

13 $$K_{p} = e^{{ - \frac{\Delta G}{RT}}}$$

where *K*_*p*_ is the equilibrium constant at constant pressure for the unfolding process. Thus, the equilibrium constant for denaturation obtained under constant pressure conditions is equal to the concentration ratio of the native and unfolded forms:

14 $$K_{p} = \frac{{[{\text{D}}]}}{{[{\text{N}}]}}.$$

## The thermodynamic basis of the HSDSC signal

The fraction of unfolded protein at temperature, *T*, multiplied by the enthalpy of unfolding at the same temperature gives the enthalpy needed to unfold *f*_*D*_ of protein at temperature, *T*. The rate of change in this enthalpy value with temperature gives the excess heat capacity—the heat capacity difference between the sample and reference cells:

15 $$C_{p,xs} = \frac{\text{d}}{{{\text{d}}T}}(f_{D} (T)\Delta H_{\text{cal}} (T)).$$

To calculate *C*_*p,xs,*_ we need to be able to calculate the changing composition of the system. This is readily done using the mass balance expression:

16 $$P_{t} = [N] + [D]$$

where *P*_*t*_ is the total concentration of protein. Rearranging Eq. 14 to provide an expression for [*N*] and substituting this in Eq. 16 give

17 $$P_{t} = [D]\left( {1 + \frac{1}{{K_{p} (T)}}} \right)$$

which can be rearranged to give the fraction of unfolded protein *f*_*D*_:

18 $$\frac{[D]}{{P_{t} }} = f_{D} = \frac{{K_{p} (T)}}{{1 + K_{p} (T)}}.$$

How do we calculate the changing composition of the system as a function of temperature? This is readily achieved using the van’t Hoff isochore [4]:

19 $$\frac{{\partial \ln (K_{p} (T))}}{\partial T} = \frac{{\Delta H_{vH} (T)}}{{RT^{2} }}$$

where Δ*H*_*vH*_(*T*) is the van’t Hoff enthalpy (for a two-state process, this is equal to the enthalpy of unfolding) and *R* is the universal gas constant. We have already noted that the unfolding process is accompanied by a positive change in heat capacity, which means that the van’t Hoff enthalpy is temperature dependent (see Eq. 7).

Substituting Eq. 7 into Eq. 19 and integration of the resultant expression give

20 $$\int\limits_{{\ln (K(T_{\text{ref}} ))}}^{\ln (K(T))} {{\text{d}}\ln (K)} = \int\limits_{{T_{\text{ref}} }}^{T} {\frac{{\Delta H_{{vH,{\text{ref}}}} + \Delta C_{p} (T - T_{\text{ref}} )}}{{RT^{2} }}} {\text{d}}T.$$

Here, Δ*H*_*vH*,ref_ is the value of the van’t Hoff enthalpy at *T*_ref_ and *T*_ref_ is the reference temperature, which is conveniently defined as the temperature at which the fractions of denatured and native protein are equal. This definition means that *K*(*T*_ref_) is equal to unity. Equation 20 is thus written, after integration, as

21 $$K(T) = K(T_{\text{ref}} )\exp \left[ {\frac{{\Delta H_{{vH,{\text{ref}}}} }}{R}\left( {\frac{1}{{T_{\text{ref}} }} - \frac{1}{T}} \right) + \frac{{\Delta C_{p} }}{R}\left( {\ln \left( {\frac{T}{{T_{\text{ref}} }}} \right) + \frac{{T_{\text{ref}} }}{T} - 1} \right)} \right].$$

Using Eq. 21 in Eqs. 18 and 16 allows us to calculate how the fractions of denatured and native protein changes vary with temperature.

## Simulating and fitting the HSDSC signal

If we complete the differentiation, as shown in Eq. 15, we obtain

22 $$C_{p,xs} (T) = \Delta H_{\text{cal}} (T)\frac{{{\text{d}}f_{D} (T)}}{{{\text{d}}T}} + f_{D} (T)\Delta C_{{p,{\text{cal}}}} .$$

In this equation, Δ*C*_*p*,cal_ is the heat capacity change obtained from the signal. We will find it convenient to differentiate between this value and the value of Δ*C*_*p*_ used in the van’t Hoff derived equations (Eqs. 20 and 21). The relationship between the two parameters is given by

23 $$\Delta C_{{p,{\text{cal}}}} = \Delta C_{p} \frac{{\Delta H_{{{\text{cal}},{\text{ref}}}} }}{{\Delta H_{{vH,{\text{ref}}}} }}.$$

To derive an analytical solution to Eq. 22, we need to find an expression for $\frac{{{\text{d}}f_{d} }}{{{\text{d}}T}}$ This is achieved using the following transformation based upon the van’t Hoff isochore (Eq. 19):

24 $$\left( {\frac{\partial \ln (K(T))}{{\partial f_{D} (T)}}} \right)_{p} \frac{{{\text{d}}f_{D} (T)}}{{{\text{d}}T}} = \frac{{\Delta H_{vH} (T)}}{{RT^{2} }}.$$

Given

25 $$K(T) = \frac{{f_{D} (T)}}{{1 - f_{D} (T)}}$$

and expressing Eq. 25 as a logarithmic expression gives

26 $$\ln (K(T)) = \ln (f_{D} (T)) - \ln (1 - f_{D} (T))$$

which on differentiation gives

27 $$\left( {\frac{{\partial {\text{In}}(K(T))}}{{\partial f_{D} (T)}}} \right)_{p} = \frac{1}{{f_{D} (T)}} + \frac{1}{{1 - f_{D} (T)}}$$

and thus provides

28 $$\frac{{{\text{d}}f_{D} (T)}}{{{\text{d}}T}} = \frac{{\Delta H_{VH} (T)}}{{RT^{2} }}\frac{1}{{f_{D} (T)}} + \frac{1}{{1 - f_{D} (T)}}.$$

The excess heat capacity can thus be written as

29 $$C_{{p,{\text{XS}}}} = \frac{{\Delta H_{\text{cal}} (T)\Delta H_{\text{vH}} (T)}}{{RT^{2} }}\frac{1}{{\frac{1}{{f_{D} (T)}} + \frac{1}{{1 - f_{D} (T)}}}} + f_{D} (T)\Delta C_{{p,{\text{cal}}}} .$$

**Fig. 5:**
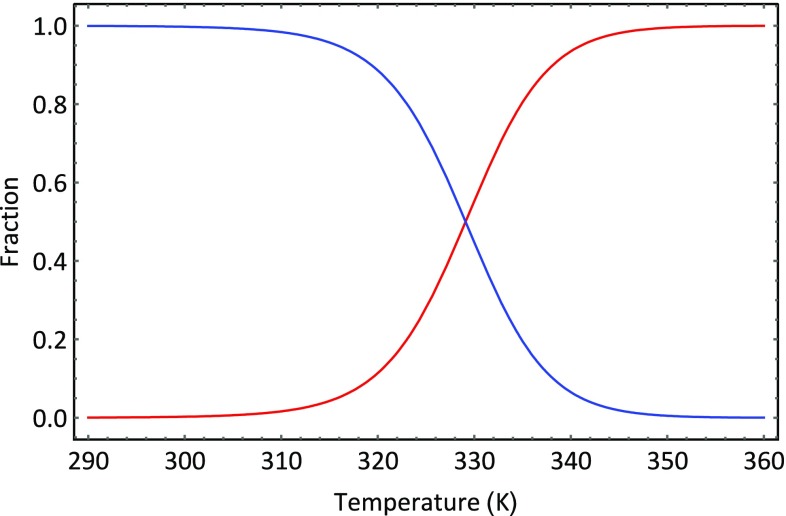
Changing composition of an aqueous protein system as a function of temperature. The fraction of molecules in the native ground state is shown in blue, and the fraction of molecules in the denatured state is shown in red. The calculation was made using the fitted parameters obtained for ubiquitin unfolding at a pH of 2

**Fig. 6:**
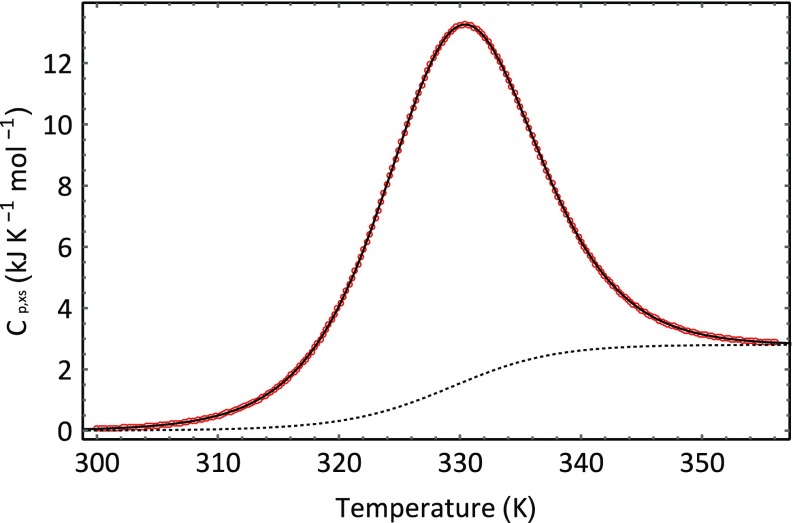
Best fit line (*inner line*) to the data (*orange open circle*) displayed in Fig. 1 using Eq. 29. The *dotted line* is the baseline given by the expression

We now have an equation for *C*_*p*,*XS*_ which is functionally related to temperature, *T*. Equation 29 can be used to fit the data shown in Fig. 1 using a least squares approach. The outcome of fitting Eq. 29 to our ubiquitin data is shown in Figs. 5 and 6. Figure 5 show how the composition of the system changes with temperature and Fig. 6 shows the optimised best fit line through the experimental data. The fitting was conducted in the following way. Initial values were assigned to the following parameters: Δ*H*_*VH*_, Δ*H*_cal_, Δ*C*_*p,*_ and *T*_ref_. These were then used in the appropriate previously defined equations to calculate an initial set of values of *C*_*p*,*XS*_ using the temperature data obtained from the data set, as shown in Fig. 1. The differences between the calculated values and the experimental values are calculated, squared, and summed. The sum of the squared differences was then minimised by changing the parameter values using the optimization routines implemented in the Computer Algebra program Mathematica (http://www.wolfram.com). For the example shown in Fig. 6, the NonlinearModelFit routine was used to fit the model to the data and provide a set of optimized parameters. The obtained optimized parameter values are shown in Tables 1 and 2. The standard errors in the parameters were very low—in the order of 0.1%.

**Table 1:** Optimized fit parameters obtained for a 5 mg cm^−3^ solution of ubiquitin in buffer solution at a pH of 2

Parameter	Estimate
Δ*H*_*VH*_	211 kJ mol^−1^
Δ*H*_cal_	198 kJ mol^−1^
*T*_ref_	329.1 K
Δ*C*_*p*_	3.0 kJ K^−1^ mol^−1^

**Table 2:** Optimized fit parameters obtained for a 5 mg cm^−3^ solution of lysozyme in 1.0 M trehalose solution

Parameter	Estimate	Standard error
Δ*H*_*vH,*ref_	418 kJ mol^−1^	2.7
Δ*H*_cal,ref_	365 kJ mol^−1^	3.2
*T*_ref_	332.4 K	0.03
*a*	−188.3 kJ mol^−1^ K^−1^	3.7
*b*	0.62 kJ mol^−1^ K^−1^	0.012
*b*	46.3 kJ mol^−1^ K^−1^	7.3
*d*	−0.08 kJ mol^−1^ K^−1^	0.02

Several observations can be made. The fitted calorimetric enthalpy is the same as the calorimetric enthalpy value measured by integration of the peak. The value of *T*_ref_ is slightly lower than the value that can be interpolated for *T*_*m*_ the temperature at which the excess heat capacity is a maximum.

The values obtained for Δ*H*_*VH*_ and Δ*H*_cal_ are close in value but not the same. This is not necessarily surprising. The units of both parameters are kJ mol^−1^. However, as we have shown, the raw calorimetric data are converted into an excess heat capacity in Eq. 2 using a mass value as measured by the experimenter. In this experiment, the required mass of protein was weighed and dissolved in buffer. The aqueous protein solution was then injected into a cell of known fixed volume. In this way, the number of moles (M) is readily calculated and inserted into Eq. 2. On the other hand, the molar unit in the van’t Hoff enthalpy is supplied by the universal gas constant. We can show this using dimensional analysis:

30 $$\left( {\frac{{\partial {\text{In}}[K_{p} (T)]}}{\partial T}} \right)_{p} = \frac{{\Delta H_{VH} (T)}}{{RT^{2} }}\mathop{\longrightarrow}\limits{{{\text{Dimensional}}\,\,{\text{analysis}}}}\frac{1}{\text{K}} = \frac{{\frac{\text{kJ}}{\text{mol}}}}{{\frac{\text{kJ}}{{{\text{mol}}\,{\text{K}}}} \times K^{2} }}.$$

It is important to note that logarithmic terms are dimensionless. Thus, in order for the equality to be true, the molar unit used in the van’t Hoff enthalpy must be identical to the molar unit supplied by the gas constant.

Therefore, why should Δ*H*_cal_ be less than Δ*H*_*VH*_? There are a number of explanations and it is indeed a matter of interest in protein thermochemistry. We make the assumption that the only thermal process on which the calorimetric enthalpy reports is the thermal unfolding of the protein. However, other thermal events may occur because of the presence of the protein in solution, which is not observed when running the baseline scans. However, it is also possible and indeed extremely likely that in our particular case not all the mass of protein placed in the cell was protein or that some of the protein placed in the cell did not undergo thermal unfolding. Both events would lead to overestimation of active protein and thus an underestimation of the calorimetric enthalpy.

We shall see later this article that there are cases, where Δ*H*_cal_ > Δ*H*_*VH*_. This arises when protein unfolding involves the appearance of substantial populations of intermediates.

There are examples, where Δ*H*_*vH*_ ≫ Δ*H*_cal_. One example is the thermally driven change from the *P*_β_ gel phase to *L*_α_ in phospholipid multi-lamellar vesicles, where the ratio of the van’t Hoff enthalpy to the calorimetric enthalpy can be as high as 200. Such numbers suggest that something like 200 phospholipid molecules are acting together as a co-operative unit.

## Protein unfolding signals when the heat capacity change is dependent upon temperature

**Fig. 7:**
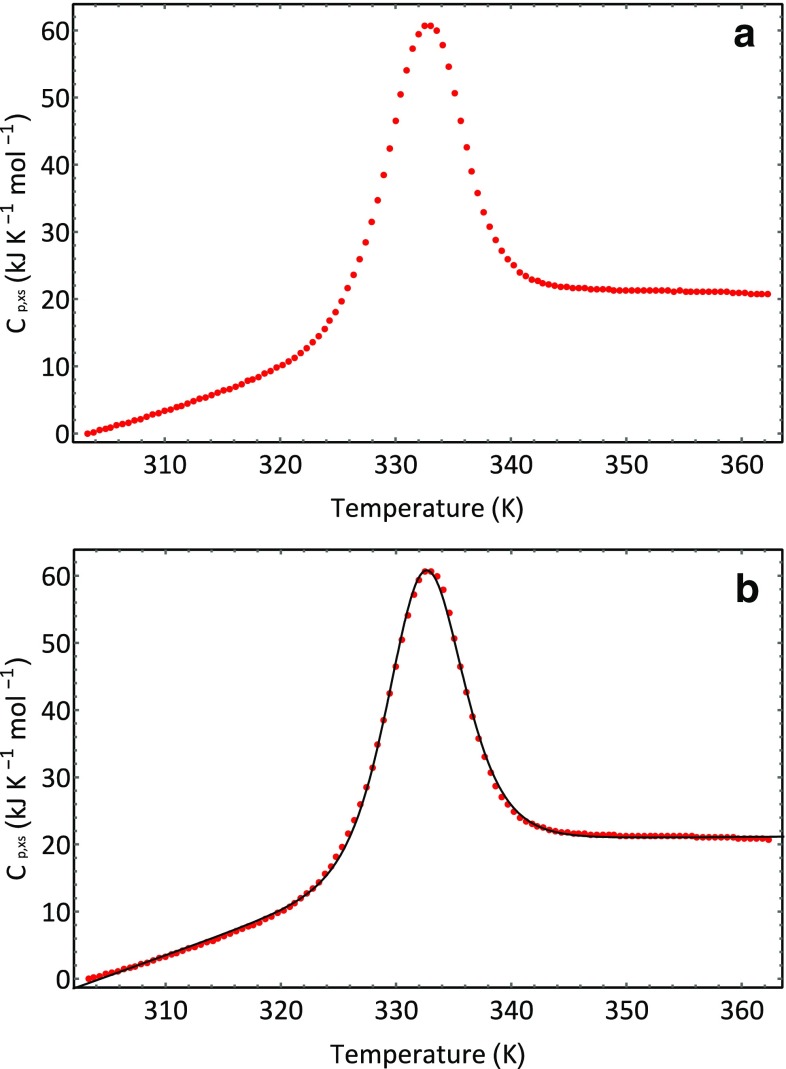
**a** HSDSC data obtained for a 5 mg cm^−3^ solution of lysozyme in 1.0 molar solution of trehalose; and **b** *line* of best fit to the data

So far, we have assumed that thermally induced unfolding is characterised by a temperature invariant heat capacity. However, it is very often the case that the pre-transitional heat capacity shows a marked functional relationship with temperature, whilst the post-transitional is somewhat flatter—less influenced by temperature. Figure 7a provides an excellent example of this kind of thermal behaviour. The signal shown was obtained in laboratory class practical for the protein lysozyme in a 1.0 M aqueous solution of trehalose. The objective of the practical was to examine the behaviour of proteins in aqueous sugar solutions. The temperature dependence of the pre- and post-transitional heat capacities of the signal is readily incorporated into our analysis.

We assume that the temperature dependence of the pre- and post-transitional heat capacities can be described by a linear relationship. The heat capacity of the native protein is given by: *C*_*p*,*N*_ = *a* + *bT,* and for the unfolded protein, *C*_*p*,*D*_ = *c* + d*T*. This provided a heat capacity change that is temperature dependent:

31 $$\Delta C_{p} (T) = (b - d)T + (c - a).$$

This is then used to provide a modified form of the Kirchhoff equation:

32 $$\left( {\frac{\partial \Delta H}{\partial T}} \right)_{p} = \Delta C_{p} (T) = (b - d)T + (c - a)\mathop{\longrightarrow}\limits{\text{Integration}} \Delta H(T) = \Delta H_{\text{ref}} + (c - a)(T - T_{\text{ref}} ) + (b - d)\left( {T - T_{\text{ref}}^{2} } \right).$$

Which then provides a modified form of Eq. 21 wherein, as before, *K*_*p*_(*T*_ref_) = 1:

33 $$\frac{{K_{p} (T)}}{{K_{p} (T_{\text{ref}} )}} = \exp \left[ \begin{aligned} \frac{{\Delta H_{{VH,{\text{ref}}}} }}{R}\left( {\frac{1}{{T_{\text{ref}} }} - \frac{1}{T}} \right) + \frac{(c - a)}{R}\left( {{\text{In}}\left( {\frac{T}{{T_{\text{ref}} }}} \right) + \frac{T}{{T_{\text{ref}} }} - 1} \right) \hfill \\ ... + \frac{(d - b)}{2R}\left( {T - T_{\text{ref}} - T_{\text{ref}}^{2} \left( {\frac{1}{{T_{\text{ref}} }} - \frac{1}{T}} \right)} \right) \hfill \\ \end{aligned} \right].$$

The temperature dependence of the heat capacity change results in the addition of another term to Eqs. 7 and 21. Equations 31, 32, and 33 can then be used in a modified form of Eq. 29 to fit the lysozyme data. The modification is made to the last term in Eq. 29 and takes into account the changing contribution of the temperature-dependent heat capacities of the folded and unfolded forms make to the underlying base line:^1^

$$\begin{aligned} f_{D} (T)C_{p,U} (T) + f_{N} (T)C_{p,N} (T) \hfill \\ \to f_{D} (T)C_{p,U} (T) + (1 - f_{D} (T))C_{p,N} (T) \hfill \\ \to f_{D} (T)\Delta C_{p} (T) + C_{p,N} (T). \hfill \\ \end{aligned}$$

Equation 29 is thus rewritten as

$$C_{p,xs} = \frac{{\Delta H_{\text{cal}} (T)\Delta H_{vH} (T)}}{{RT^{2} }}\frac{1}{{\frac{1}{{f_{D} (T)}} + \frac{1}{{1 - f_{D} (T)}}}} + \frac{{\Delta H_{\text{cal}} }}{{\Delta H_{vH} }}(f_{D} (T)\Delta C_{p} (T) + C_{p,N} (T)).$$

**Fig. 8:**
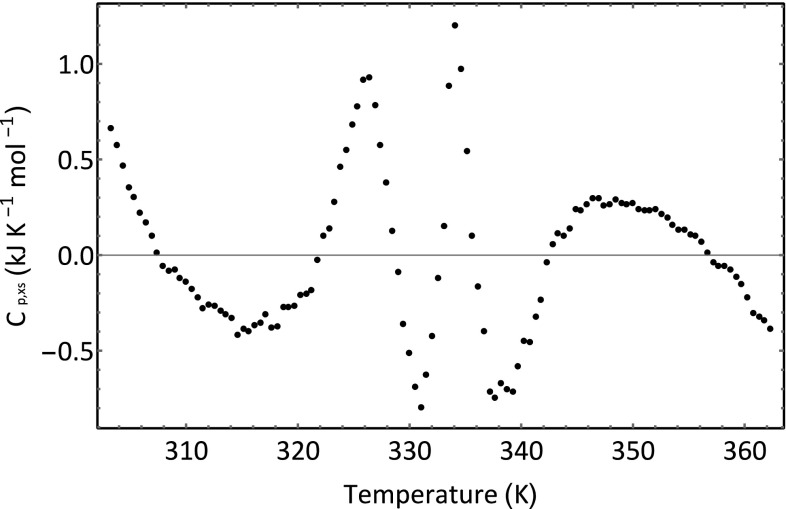
Residual plot for the fit shown in Fig. 7b

It will be recalled that the calorimetric enthalpy van’t Hoff enthalpy ratio appears to appropriately scale the contribution of the underlying baseline to calorimetric signal. This modified form of Eq. 29 was fitted to data obtained for an aqueous solution of lysozyme in 1 M trehalose solution. The solution concentration was 5 g dm^−3^. The results of this fit are shown in Fig. 7b. The optimised fit parameters are displayed in Table 2. The adjusted *R* ^2^ value for the fit is 0.999; the following optimized fit parameters were obtained. Both the adjusted *R* ^2^ value and Fig. 7b seem to suggest that the fit is extremely good. However, it is always good practice to look at a plot of the residuals. The residuals are calculated as the difference between the measured value for *C*_*p,xs*_ and the calculated value *C*_*p,xs*_ using the best fit parameters. A residual plot is shown in Fig. 8. If the residuals arise purely from the uncertainties in measurement—for example, instrumental noise, then it would be expected that the residuals would be located at random about the *C*_*p,xs*_ axis. The fact that they are not reveals that there are systematic errors either in the data or in an error(s) in the model used to fit the data. Most probably, the analysis has neglected some other minor thermal events.

Of further note is the rather large discrepancy between the calorimetric and van’t Hoff enthalpies. The calorimetric enthalpy is 87.3% of the value of the van’t Hoff enthalpy. The lysozyme sample used was obtained from Sigma Aldrich who claims that its purity is ≥90%. For the experiment, the lysozyme was used as received which may thus explain the discrepancy.

## Multiple transitions

The residual plot in Fig. 8 suggests that the model used to fit the data may have been too simple in that other thermal events may also occur which have not been incorporated into the model. If these thermal events are independent of the main transition, then the overall thermal transition is a simple arithmetic addition of the two underlying events. For example, small amounts of an impurity, which is also calorimetrically observable, may be present in the sample. It is also possible that in the case of a multi-sub-unit protein, the sub-units unfold independently of each other. Again, the thermal signal would be a composite of the underlying transitions. However, protein unfolding may involve the native protein undergoing a transition to one or several intermediate states before ultimately adopting the final unfolded form. Protein unfolding, under equilibrium conditions, by such a mechanism can be represented by the following mass action expression in the case, where there are two intermediates formed in significant quantities:

$$N\overset {K_{1} } \leftrightarrows I_{1} \overset {K_{2} } \leftrightarrows I_{2} \overset {K_{3} } \leftrightarrows D.$$

To be able to calculate the fraction of each species at any particular temperature, *T*, we formulate the following mass balance expression:

34 $$P_{t} = [N] + [I_{1} ] + [I_{2} ] + [D]$$

where *P*_*t*_ is the total protein concentration and [] terms represent the equilibrium concentrations of the respective species. If we divide Eq. 34 by the [*N*] and invert, we obtain the following expression for the fraction of native protein:

35 $$\frac{[N]}{{P_{t} }} = \alpha_{N} = \frac{1}{{1 + \frac{{[I_{1} ]}}{[N]} + \frac{{[I_{2} ]}}{[N]} + \frac{[D]}{[N]}}}.$$

Because unfolding occurs under equilibrium conditions, we can write the following equilibrium equations:

36 $$K_{1} (T) = \frac{{[I_{1} ]}}{[N]}\,\,K_{2} (T) = \frac{{[I_{2} ]}}{{[I_{1} ]}}\,\,K_{3} (T) = \frac{[D]}{{[I_{2} ]}}.$$

Using these expressions in Eq. 35, we obtain

37 $$\alpha_{N} (T) = \frac{1}{{1 + K_{1} (T) + K_{1} (T)K_{2} (T) + K_{1} (T)K_{2} (T)K_{3} (T)}}.$$

Similar expression is readily derived for the fractions of the other intermediate and denatured species:

38 $$\begin{aligned} &\alpha_{1} (T) = K_{1} (T)\alpha_{N} (T) \hfill \\ & \alpha_{2} (T) = K_{1} (T)K_{2} (T)\alpha_{N} (T) \hfill \\ & \alpha_{D} (T) = K_{1} (T)K_{2} (T)K_{3} (T)\alpha_{N} (T). \hfill \\ \end{aligned}$$

**Fig. 9:**
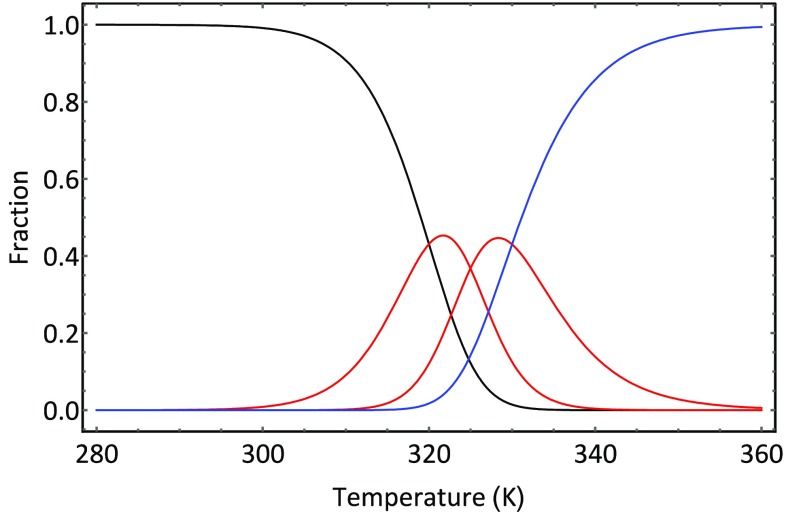
*Graph* showing how the fraction of protein species varies with temperature using the model data in Table 3

The equilibrium constants are calculated using Eq. 21. Given the following model thermodynamic data, the fractional composition of an aqueous protein solution is depicted in Fig. 9.

Simulating the HSDSC signal using the parameters in Tables 3 and 4 is slightly more complicated than our previous examples. The greater complexity comes from correctly identifying the enthalpy changes that accompany the formation of each species. Essentially, all enthalpy changes are calculated with the native form as the low energy form of the protein. Thus, the enthalpy change accompanying the formation of *I*_1_ is Δ*H*_*VH*,1_; the enthalpy change accompanying the formation of *I*_2_ is Δ*H*_*VH*,1_ + Δ*H*_*VH*,2;_ and the enthalpy of denaturation is given by Δ*H*_*VH*,1_ + Δ*H*_*VH*,2_ + Δ*H*_*VH*,3_. Thus, the following expression can be then be used to calculate the excess heat capacity assuming Δ*H*_cal_ = Δ*H*_*vH*_:

39 $$\begin{aligned} C_{p,XS} = \Delta H_{VH,1} (T)\frac{{{\text{d}}\alpha_{1} (T)}}{{{\text{d}}T}} + \alpha_{1} (T)\Delta C_{P,1} \, + \hfill \\ ... (\Delta H_{VH,1} (T) + \Delta H_{VH,2} (T))\frac{{{\text{d}}\alpha_{2} (T)}}{dT} + \alpha_{2} (T)(\Delta C_{P,1} + \Delta C_{P,2} ) \, + \hfill \\ ... (\Delta H_{VH,1} (T) + \Delta H_{VH,2} (T) + \Delta H_{VH,3} (T))\frac{{{\text{d}}\alpha_{3} (T)}}{{{\text{d}}T}} + \alpha_{3} (T)(\Delta C_{P,1} + \Delta C_{P,2} + \Delta C_{P,3} ). \hfill \\ \end{aligned}$$

If we collect together the appropriate terms, then Eq. 39 can be written in terms of the underlying component transitions as follows:

40 $$\begin{aligned} C_{p,xs} = \Delta H_{vH,1} (T)\left( {\frac{{{\text{d}}\alpha_{1} (T)}}{{{\text{d}}T}} + \frac{{{\text{d}}\alpha_{2} (T)}}{{{\text{d}}T}} + \frac{{{\text{d}}\alpha_{3} (T)}}{{{\text{d}}T}}} \right) + \left( {\alpha_{1} (T) + \alpha_{2} (T) + \alpha_{3} (T)} \right)\Delta C_{p,1} + \\ ... \Delta H_{vH,2} \left( T \right)\left( {\frac{{{\text{d}}\alpha_{2} (T)}}{{{\text{d}}T}} + \frac{{{\text{d}}\alpha_{3} (T)}}{{{\text{d}}T}}} \right) + \left( {\alpha_{2} (T) + \, \alpha_{3} (T)} \right)\Delta C_{p,2} + \\ ... \Delta H_{vH,3} (T)\frac{{{\text{d}}\alpha_{3} (T)}}{{{\text{d}}T}} + \alpha_{3} (T)\Delta C_{p,3} . \\ \end{aligned}$$

The derivatives in Eqs. 39 and 40 can be estimated using a centred finite difference approximation:

41 $$\frac{{{\text{d}}\alpha (T)}}{{{\text{d}}T}} \, \approx \, \frac{\alpha (T + \delta T) - \alpha (T - \delta T)}{2\delta T}.$$

**Fig. 10:**
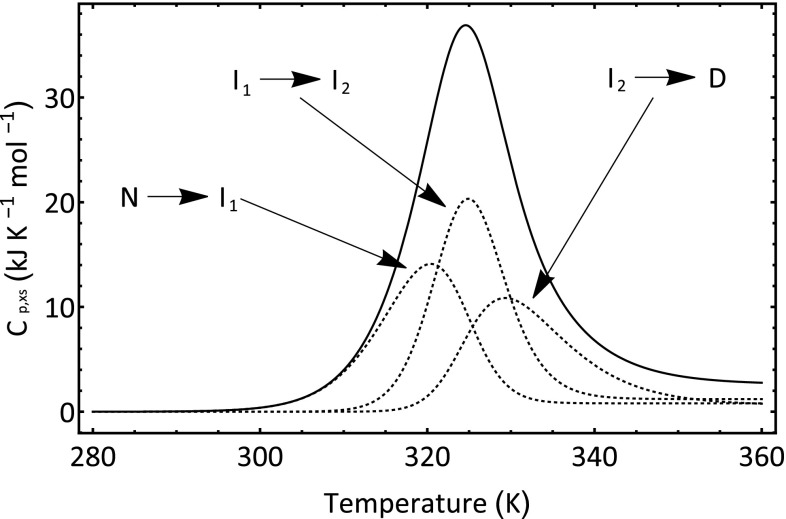
Simulated DSC signal for a model protein system wherein unfolding involves the formation of two intermediates. The component transitions are identified and shown. A value of was used in the simulation

**Fig. 11:**
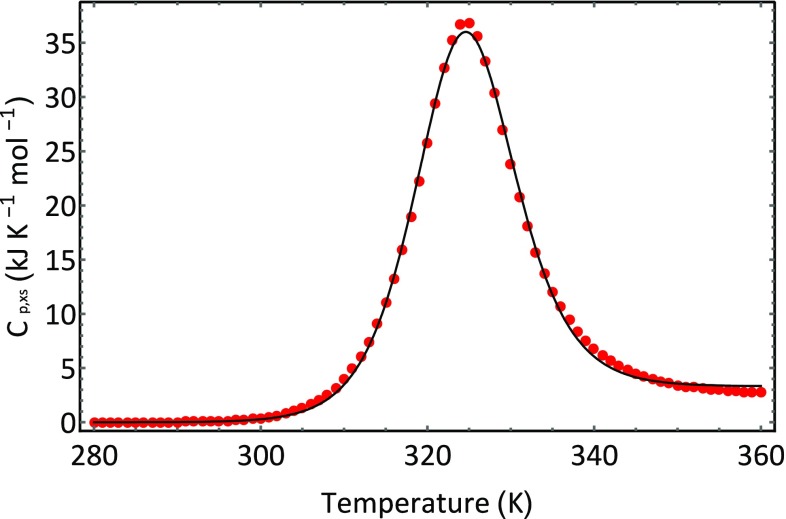
Fit of the overall simulated signal shown in Fig. 10 to a two-state model

The simulated DSC signal using the data in Table 3 and Eqs. 21, 37, 38, 39, 40, and 41 is shown in Fig. 10. It is worth nothing that the shapes of the component transitions are not symmetrical. The overall thermal transition can be fitted to a two-state model as was the data obtained for ubiquitin. This is shown in Fig. 11. The fit is not especially poor and could lead inexperienced experimenters to conclude that the transition is two states. However, the optimized fit parameters show immediately that the use of the two-state model is incorrect. The van’t Hoff enthalpy value is 218 kJ mol^−1^, whilst the calorimetric enthalpy is 548 kJ mol^−1^. As we pointed previously in the manuscript, if Δ*H*_cal_ > Δ*H*_*vH,*_ then the presence of significant populations of intermediate states in the transition is inferred.

**Table 3:** Model data used to show the effect of temperature upon the composition of an aqueous protein solution using Eqs. 21, 37, and 38

Transition	Δ*H*_*VH*_	T_ref_	Δ*C*_*p*_
1	190	320	0.8
2	220	325	1.2
3	170	330	0.6

**Table 4:** Parameter values used to simulate the HSDSC signal shown in Fig. 12

Parameter	Assumed value
Δ*H*_*vH*,ref_	300 kJ mol^−1^
Δ*H*_cal,ref_	295 kJ mol^−1^
*T*_ref_	330 K
*a*	−30 kJ mol^−1^ K^−1^
*b*	0.11 kJ mol^−1^ K^−1^
*b*	9.0 kJ mol^−1^ K^−1^
*d*	0.01 kJ mol^−1^ K^−1^

## Transitions involving dissociation

Many proteins have a quaternary structure which involves the association of several folded molecular sub-units to form a multiple sub-unit complex. The simplest complex is a dimer. One such example, examined by Sturtevant and co-workers, is the tryptophan repressor obtained from *Escherichia Coli*, which shows unusual thermal stability at pH 7.5 [8]. Their DSC traces show that the heat capacity change is temperature dependent and that the peak itself shows a significant amount of asymmetry. Furthermore, the signal shows some concentration dependence. To develop a thermodynamic model that can encapsulate these observations, we need to use Eq. 33 that incorporates the temperature effects upon heat capacity. However, we need to be extremely careful about the equilibrium constant equations that we use. We shall find it expedient to formulate these equations in terms of the fraction of protein that has undergone dissociation/denaturation.

We assume that the following equilibrium equation adequately describes the dissociation/unfolding process:

$${\text{N}}_{2} \rightleftharpoons 2{\text{D}} .$$

In other words, dissociation and unfolding occur at more or less the same time. Or to use the language, we used in the previous section on multiple transitions the population of dissociated sub-units in the native form is extremely if not vanishingly small.

To make the simulations simple, we shall assume that both sub-units comprising the dimer are the same, so that we can use the following mass action description of the equilibrium process:

$$K_{p} (T) = \frac{{[D]^{2} }}{{[N_{2} ]}}.$$

If *P*_*t*_ is the total concentration of the dimeric protein, then we can write the following:

42 $$[D] = 2f_{D} P_{t} .$$

Here, *f*_*D*_ is the fraction of dimer that has undergone dissociation/denaturation. Similarly

43 $$[N_{2} ] = (1 - f_{D} )P_{t} .$$

The equilibrium constant is thus written as

44 $$K_{p} (T) = \frac{{[D]^{2} }}{{[N_{2} ]}} = \frac{{4f_{D}^{2} P_{t} }}{{(1 - f_{D} )}}.$$

We now need to define *K*(*T*_ref_) *T*_ref_ is the temperature at which half the protein has undergone dissociation/denaturation, i.e., *f*_*D*_ = 0.5. We shall, however, also define *T*_ref_ in terms of a reference concentration *P*_ref:_

45 $$K_{p} (T_{\text{ref}} ) = \frac{{4 \times 0.5^{2} \times P_{\text{ref}} }}{(1 - 0.5)} = 2P_{\text{ref}} .$$

Substituting Eqs. 44 and 45 into Eq. 33 gives

46 $$\frac{{2f_{D}^{2} }}{{(1 - f_{D} )}}\frac{{P_{t} }}{{P_{\text{ref}} }} = \exp \left[ \begin{aligned} \frac{{\Delta H_{{VH,{\text{ref}}}} }}{R}\left( {\frac{1}{{T_{\text{ref}} }} - \frac{1}{T}} \right) + \left( {\frac{c - a}{R}} \right)\left( {{\text{In}}\frac{T}{{T_{\text{ref}} }}} \right) + \frac{{T_{\text{ref}} }}{T} - 1 \hfill \\ ... + \frac{(d - b)}{2R}\left( {T - T_{\text{ref}} - T_{\text{ref}}^{2} \left( {\frac{1}{{T_{\text{ref}} }} - \frac{1}{T}} \right)} \right) \hfill \\ \end{aligned} \right].$$

Equation 46 is a quadratic expression in terms of *f*_*D*_. As before, if we define all the parameters on the right-hand side (the values are shown below) as well as the concentrations, we can calculate *f*_*D*_ using the normal solution for quadratic equations. These values are then used in Eq. 29 to simulate the HSDSC signal.

**Fig. 12:**
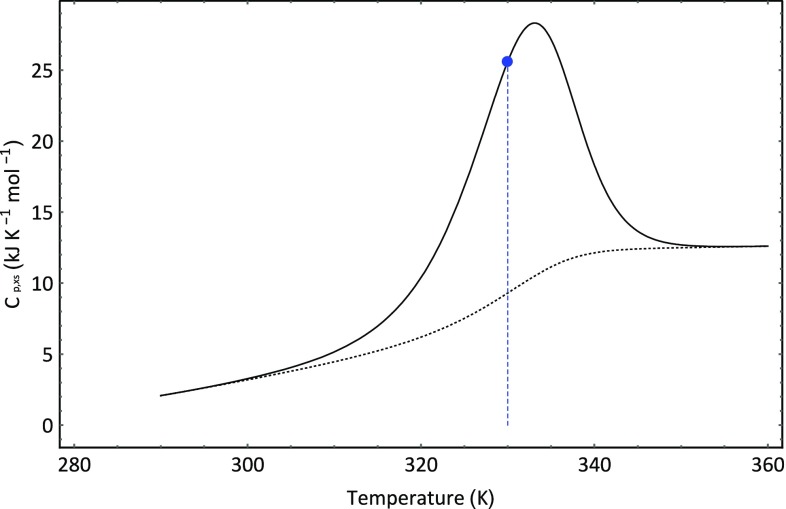
Simulation of dissociative unfolding of a dimer protein complex. The blue line shows the location of *T*_ref_ the temperature at which half the protein has undergone the thermal transition

**Fig. 13:**
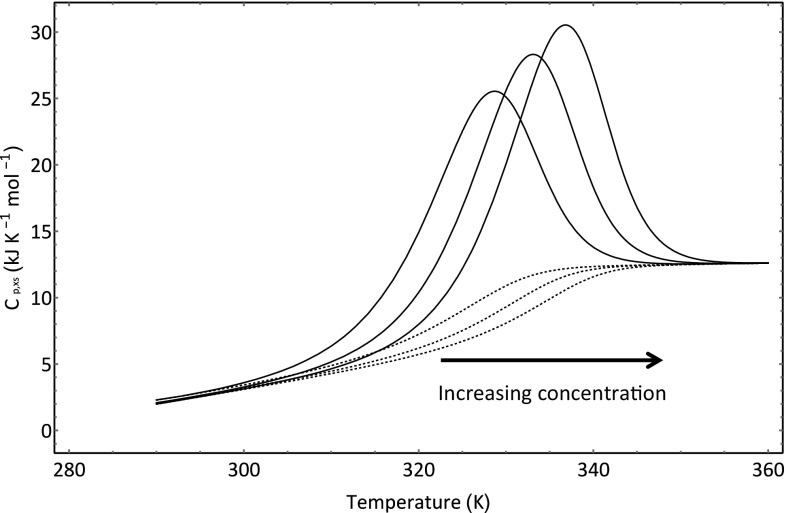
Simulation showing the effect of concentration upon thermal transition shown in Fig. 12

For the simulation of the HSDSC signal shown in Fig. 12, the data displayed in Table 4 was used and it was assumed that *P*_*t*_ = *P*_ref_. Comparison between the data provided by Sturtevant et al. [8] and Fig. 13 shows that the simulation captures the major features of the experimental data. The heat capacity is temperature dependent, and the signal shows distinct asymmetry. Moreover, in Figs. 12 and 13, it is shown that *T*_ref_ does not correspond to the temperature of maximum excess heat capacity. If we change the protein concentration, then we can show that the signal shifts to higher temperature ranges when the concentration is increased and to lower temperature ranges when the concentration is lowered in line with experimental observations [8]. The observant reader will no doubt detect that the signals appear larger at higher concentrations. This is to be expected, since the transitions occur over higher temperature ranges at higher protein concentrations and the positive heat capacity change thus results in increases in both the calorimetric and van’t Hoff enthalpies.

## Concluding remarks

The aim of this article has been to examine the thermodynamics of temperature induced changes in aqueous protein systems as detected by scanning calorimetry. I have tried to show how through the use of simple models of protein unfolding and through the application of familiar thermodynamic relationships, the scanning calorimetric signals can be simulated and fitted to these models. The text, however, does come with a caveat. Calorimetric signals can be, and very often are, over interpreted. The model selected must fit the known attributes of the thermally induced transition. It is not uncommon to see novices try to fit a dissociation transition (that always shows a distinct asymmetric peak) to a model that involves several independent transitions using the software supplied by the instrument manufacturer. The better the novice understands, the underpinning science of signal creation the more likely they shall be able to correctly interpret that signal.

## Footnotes

1 In the treatment of ubiquitin the heat capacity of the native form was arbitrarily set to zero after the subtraction of the instrumental baseline and so does not appear in Eq. 29.
